# Influenza infections in Australia 2009–2015: is there a combined effect of age and sex on susceptibility to virus subtypes?

**DOI:** 10.1186/s12879-019-3681-4

**Published:** 2019-01-10

**Authors:** Kam Cheong Wong, Georgina M. Luscombe, Catherine Hawke

**Affiliations:** 1 Bathurst Rural Clinical School, School of Medicine, Western Sydney University, Bathurst, NSW Australia; 2 School of Rural Health, Faculty of Medicine and Health, University of Sydney, Orange, NSW Australia; 3 Westmead Applied Research Centre, Faculty of Medicine and Health, University of Sydney, Sydney, NSW Australia

**Keywords:** Influenza surveillance, Influenza virus subtype, Influenza virus type A, Influenza virus type B, Age, Sex

## Abstract

**Background:**

Influenza is a global infectious disease with a large burden of illness and high healthcare costs. Those who experience greater burden of disease include younger and older people, and pregnant women. Although there are known age and sex susceptibilities, little is known about how the interaction of age and sex may affect a population’s vulnerability to infection with different subtypes of influenza virus.

**Methods:**

Laboratory-confirmed cases of influenza notified between 1 January 2009 and 31 December 2015 obtained from the Australian Government National Notifiable Diseases Surveillance System Influenza Public Data Set were analysed by age, sex and virus subtype. Age standardised notification rates per 100,000 population were calculated separately for females and males and used to generate female-to-male ratios with 95% confidence intervals for influenza A and B, and for virus subtypes A(H1N1)pdm09 and A(H3N2).

**Results:**

334,560 notifications for influenza A (all notifications), A(H1N1)pmd09, A(H3N2) and B subtypes from a total of 335,414 influenza notifications were analysed. Male notification rates were significantly higher for the 0 to 4 years old age group regardless of virus type or subtype; and higher for those aged 0 to 14 years and those 85 years and older for influenza types A and B and subtype A(H1N1)pdm09. Female notification rates were significantly higher for A(H1N1)pdm09 in those aged 15 to 54 years, for Type A and sub-type A(H3N2) in those aged 15 to 69 years, and for Influenza B in those aged 20 to 74 years.

**Conclusions:**

We observed a female dominance in notification rates throughout the adult age groups, which could possibly be related to health seeking behaviours. However, differences in health seeking behaviours cannot explain the variations observed across virus subtypes in the particular age groups with higher female notifications. Depending on their age, females may be more susceptible to certain subtypes of influenza virus. These observations suggest that there is an interaction between age and sex on susceptibility to influenza infection which varies by the subtype of the virus. The inclusion of pregnancy and menopausal status in surveillance data may assist development of targeted public health approaches during the emergence of new subtypes of influenza virus. Targeted vaccination campaigns may need to take into consideration specific age and sex groups who have a greater susceptibility to influenza infection as well as those who experience a greater burden of illness.

## Background

Influenza is a global infectious disease that affects human populations through epidemics and pandemics. The World Health Organization (WHO) estimates that annual influenza epidemics result in approximately 3 to 5 million cases of severe illness with a large burden of disease including between 250,000 and 500,000 deaths [1], and substantial healthcare costs [2, 3]. The burden is greatest for those at risk of influenza related complications i.e. younger and older people and women who are pregnant or post-partum [1, 4]. These observations allude to the presence of age and sex differences in influenza infections. Age variations in the rates of different subtypes of influenza infections have been reported in several studies [5–9]. Sex disparities in the infection have not been studied extensively [10] but they may exist due to the difference in hormonal and immune responses to the infection [10–17]. Giefing-Kroll and colleagues (2015) reported sex disparities in influenza infections, with premenopausal women susceptible to pandemic influenza and men to seasonal influenza. They note that variations by sex and virus subtype across countries remain to be resolved.

Most of the reports on age and sex disparities pertain to the A(H1N1) pandemic. For example, Jacobs and colleagues (2012) observed sex differences in the risk of pandemic A(H1N1) virus by age, with post-menopausal women having a lesser rate of decline in risk of infection compared with men of the same age. The authors noted a scarcity of information on the influence of sex specific hormones on disease susceptibility and severity [18]. A WHO report summarised sex differences using data from the first wave of the 2009 H1N1 pandemic [19] and concluded that the outcome of infection was generally worse for females. The 13 studies included in the WHO report, which varied in size between 43 to 8381 cases, generally reported on influenza outcomes rather than susceptibility. Furthermore, female to male risk ratios were not reported for any specific age group [19]. WHO acknowledged that the interaction between age and sex in influenza infection deserves more attention among researchers and policy-makers. In the same vein, Klein and Pekosz (2014) have suggested that sex-specific vaccines should be considered [17].

Despite acknowledgement that sex may influence both susceptibility and response to influenza infection, studies examining sex variations in age-specific notification rates of different subtypes of influenza virus are lacking. The objective of our study was to investigate age and sex interactions in laboratory-confirmed influenza notifications by subtype of the virus.

## Methods

We obtained the Australian Government National Notifiable Diseases Surveillance System Influenza (NNDSSI) Public Data Set (http://www9.health.gov.au/cda/source/pub_influ.cfm; accessed on 10th October 2016) which includes laboratory-confirmed cases of influenza notified between 1st January 2009 and 31st December 2015 (inclusive) [20]. The variables in the dataset included week of notification, location of testing (Australian state or territory), influenza type and subtype, age group at onset (in 5-year bands), sex (male, female, indeterminate/ intersex/ unspecified, unknown), and Indigenous status (Indigenous, non-Indigenous, unknown). Estimated resident populations (ERP), by age, sex and year, for the timeframe were sourced from the Australian Bureau of Statistics [21]. Notification rates per 100,000 population were generated using these ERPs, both for the whole population and for males and females separately. These age standardised notification rates were calculated both for the entire surveillance period (2009 to 2015) and for each calendar year for Influenza types (A or B), and for each of the Influenza A subtypes. Relative notification rate ratios (female-to-male ratios) with 95% confidence intervals [22] were used to compare notification between sexes (with values greater than 1 indicating a female preponderance). Due to known regional differences in subtyping, a sensitivity analysis was performed exploring relative rate ratios by location for those states comprising at least 85% of Influenza A and B notifications and subtypes A(H1N1)pdm09 and A(H3N2). All analyses were conducted using IBM SPSS v24 and *p* < 0.05 was deemed to indicate statistical significance.

## Results

From January 2009 to December 2015 there were 335,414 influenza notifications, with influenza B and A(H1N1)pdm09 the predominant viruses (see Table 1). Overall, 38.3% of the 236,820 influenza A notifications were subtyped. In 2009, 11.2% (6488) of the total 57,738 notifications were for the non-pandemic subtype i.e. A(H1N1). During the seven influenza seasons, notifications for A(H1N1)pdm09 decreased and for influenza B increased. Of the 334,560 notifications for influenza types A and B, 53.0% were females (*n* = 177,219), 0.2% (*n* = 594) were indeterminate, intersex or unknown and 49.9% were aged 29 years or younger. The sex distribution varied by virus subtype (female: 52.0% A(H1N1 pdm09), 54.5% A(H3N2) and 52.6% influenza B, *p* < 0.001) as did the age distribution (29 years or younger: 59.6% A(H1N1)pdm09, 41.3% A(H3N2) and 56.1% influenza B, p < 0.001).

**Table 1:** Summary of Influenza notifications, by year and by virus type and subtype, Australia, 2009–2015

Subtype	TOTAL	2009	2010	2011	2012	2013	2014	2015
All notifications	335,414	57,738	13,368	26,952	43,846	27,629	66,456	99,425
Influenza A	236,820 (70.8%)	57,147 (99.2%)	11,994 (90.2%)	19,544 (72.9%)	33,374 (76.3%)	17,355 (63.0%)	58,391 (88.0%)	39,015 (39.3%)
A(H1N1)pdm09	58,618 (17.5%)	30,706 (53.2%)	7515 (56.2%)	7058 (26.2%)	305 (0.7%)	3958 (14.3%)	6813 (10.3%)	2263 (2.3%)
A(H3N2)	25,224 (7.5%)	1685 (2.9%)	542 (4.1%)	1996 (7.4%)	7374 (16.8%)	1620 (5.9%)	5532 (8.3%)	6475 (6.5%)
Influenza B	97,740 (29.2%)	453 (0.8%)	1307 (9.8%)	7247 (27.1%)	10,394 (23.7%)	10,199 (37.0%)	7940 (12.0%)	60,200 (60.7%)

Footnote: notifications that were un-typed (*n* = 263), and co-infections (*n* = 566) or uncommon Influenza viruses (e.g. A(H1N1) *n* = 6488; influenza C *n* = 25) are not presented

Source: NNDSSI, extracted on 10th October 2016

### Age patterns by virus subtype

**Fig. 1:**
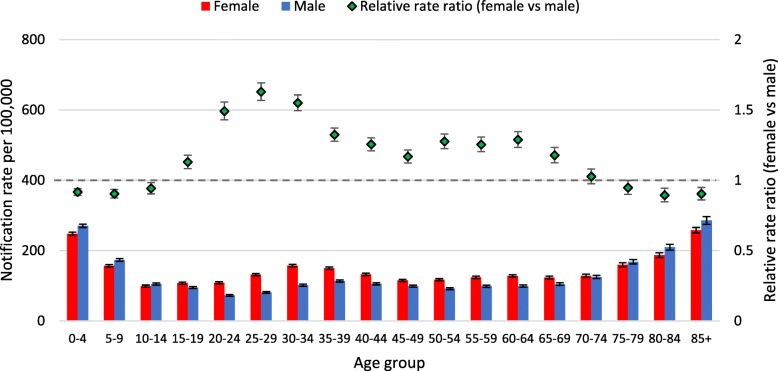
Age-specific notifications per 100,000 population of Influenza A (all notifications) and relative rate ratio in Australia 2010–15 (*N* = 179,447). Note: the dotted grey line is the reference line for a relative rate ratio of 1 (equivalent female to male rate), ratio >1 indicates female preponderance

**Fig. 2:**
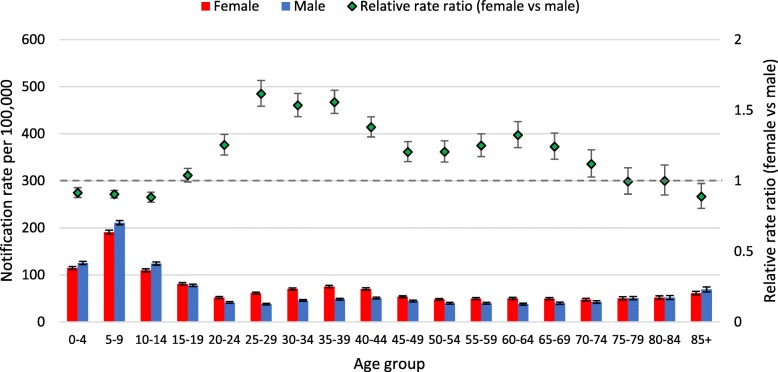
Age-specific notifications per 100,000 population of Influenza B and relative rate ratio in Australia 2010–2015 (*N* = 97,214). Note: the dotted grey line is the reference line for a relative rate ratio of 1 (equivalent female to male rate), ratio >1 indicates female preponderance

**Fig. 3:**
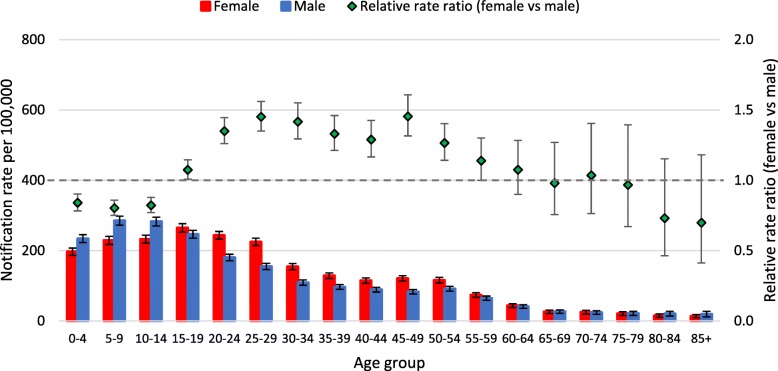
Age-specific notifications per 100,000 population of A(H1N1)pdm09 and relative rate ratio in Australia 2009 (*N* = 30,567). Note: the dotted grey line is the reference line for a relative rate ratio of 1 (equivalent female to male rate), ratio >1 indicates female preponderance

**Fig. 4:**
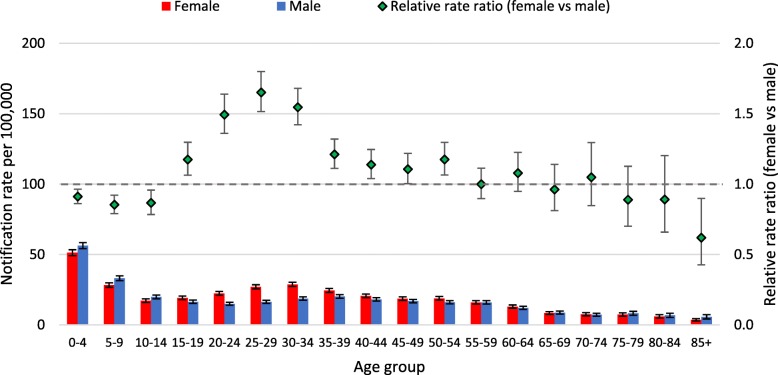
Age-specific notifications per 100,000 population of A(H1N1)pdm09 and relative rate ratio in Australia 2010–2015 (*N* = 27,883). Note: the dotted grey line is the reference line for a relative rate ratio of 1 (equivalent female to male rate), ratio >1 indicates female preponderance

**Fig. 5:**
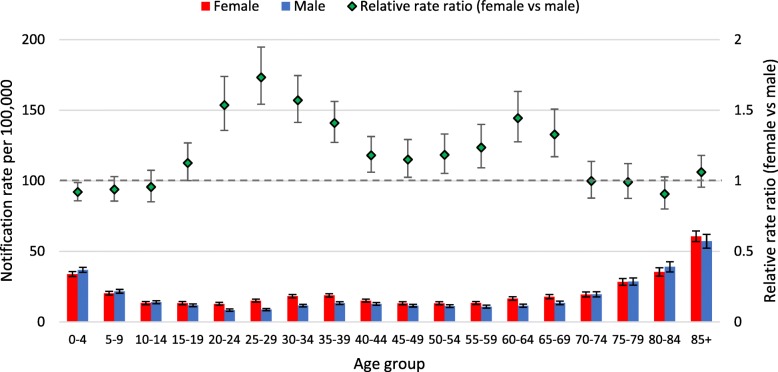
Age-specific notifications per 100,000 population of A(H3N2) and relative rate ratio in Australia 2010–2015 (*N* = 23,531). Note: the dotted grey line is the reference line for a relative rate ratio of 1 (equivalent female to male rate), ratio >1 indicates female preponderance

Different patterns in notification rates per 100,000 by age were observed for the two virus types (Figs. 1, 2) and the Influenza A virus subtypes (Figs. 3, 4, 5). Influenza subtype A(H1N1)pdm09 was more common in younger cohorts. However, when age patterns were reviewed for 2009 pandemic year alone the peak notification was for 5 to 19 years old, whereas for the post-pandemic period (2010 to 2015) peak notification rate was for 0 to 4 years old (Fig. 3, 4). The age-specific notification rate of A(H3N2) appeared as a “U-shaped curve” i.e. higher notifications among the young and elderly (Fig. 5), similar to Influenza A (all notifications). The notification rate of influenza B also showed a predominance in the younger age group, with a marked peak among 5 to 9 years old (Fig. 2).

### Age and sex patterns by virus subtype

**Fig. 6:**
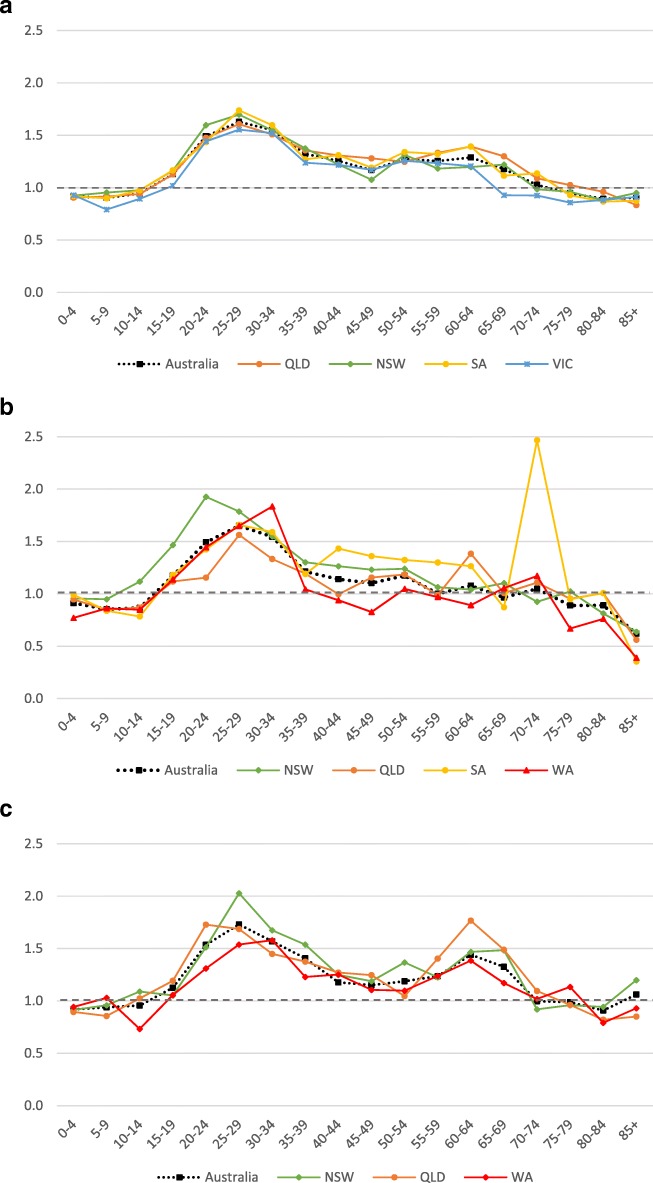
**a** Relative rate ratio for Influenza A (all notifications), by state in Australia 2010–15 (N = 179,447) (**b)** Relative rate ratio for Influenza A(H1N1)pdm09, by state in Australia 2010–15 (N = 27,858) (**c)** Relative rate ratio for Influenza A(H3N2), by state in Australia 2010–15 (N = 23,516) QLD, Queensland; NSW, New South Wales; SA, South Australia; VIC, Victoria. Note: the dotted grey line is the reference line for a relative rate ratio of 1 (equivalent female to male rate), ratio >1 indicates female preponderance. Queensland, New South Wales, South Australia and Victoria comprise 88% of Influenza A notifications across Australia during 2010-15.

**Fig. 7:**
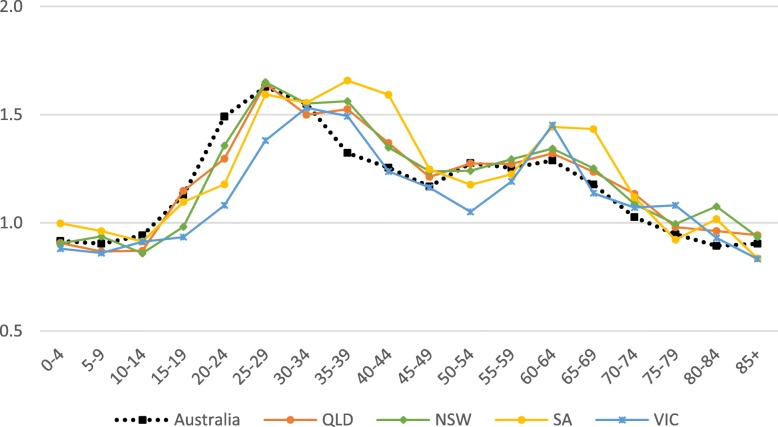
Relative rate ratio for Influenza B, by state in Australia 2010–15 (N = 97,214). NSW, New South Wales; QLD, Queensland; SA, South Australia; WA, West Australian. Note: the dotted grey line is the reference line for a relative rate ratio of 1 (equivalent female to male rate), ratio >1 indicates female preponderance. New South Wales, Queensland, South Australia and Western Australia comprise 87% of Influenza A(H1N1)pdm09 notifications across Australia during 2010-15. Note, the anomalous data point for 70-74 years old for South Australia is a consequence of small notification numbers (n=33).

The patterns of female or male preponderance by age for all Influenza A and B virus notifications for 2010–15 look similar, with a male preponderance for younger and older cases, and a female preponderance during the middle years (see Fig. 1, 2). These trends were relatively consistent for Influenza A and B notifications between different states (see Fig. 6a to c, Fig. 7). When considering all notifications, regardless of year of notification, we observed significantly higher female-to-male ratios from ages 20 to 54 years (see Table 2). This pattern differed between types and subtypes, with higher notification rates for females compared to males for 15 to 69 years for Influenza A, 15 to 54 years for A(H1N1)pdm09, 15 to 69 years for A(H3N2), and 20 to 74 years for influenza B. Male notification rates were significantly higher for the 0 to 4 years old age group regardless of virus type or subtype; and higher for those aged 0 to 14 years and those 85 years and older for influenza A, influenza A(H1N1)pdm09 and influenza B.

**Table 2:** Notification rates per 100,000 and relative rate ratios (female-to-male ratio) by virus type and subtype, Australia 2009–2015

	A (all notifications) *N* = 236,171			A(H1N1)pdm09 *N* = 58,425			A(H3N2) *N* = 25,147			B *N* = 97,646		
	Notification rate		Relative rate ratio	Notification rate		Relative rate ratio	Notification rate		Relative rate ratio	Notification rate		Relative rate ratio
Age group	Male	Female	(95% CI)	Male	Female	(95% CI)	Male	Female	(95% CI)	Male	Female	(95% CI)
0–4	292.0	262.7	.90 (.88–.92) ^a^	80.6	71.1	.88 (.84–.92) ^a^	34.1	31.1	.91 (.85–.98) ^a^	108.4	99.3	.92 (.88–.95) ^a^
5–9	221.7	195.2	.88 (.86–.91) ^a^	67.1	55.4	.83 (.78–.87) ^a^	20.4	19.3	.95 (.87–1.03)	182.8	165.3	.90 (.88–.93) ^a^
10–14	168.9	151.7	.90 (.87–.93) ^a^	57.2	47.7	.83 (.79–.88) ^a^	14.2	13.4	.94 (.85–1.05)	107.0	94.5	.88 (.85–.92) ^a^
15–19	150.1	160.0	1.07 (1.03–1.10) ^b^	49.2	54.3	1.10 (1.05–1.16) ^b^	11.0	12.6	1.14 (1.02–1.28) ^b^	67.2	69.9	1.04 (.99–1.09)
20–24	107.0	149.4	1.40 (1.35–1.44) ^b^	38.1	53.0	1.39 (1.32–1.47) ^b^	7.8	12.2	1.55 (1.38–1.74) ^b^	36.0	45.3	1.26 (1.19–1.33) ^b^
25–29	106.9	163.1	1.53 (1.48–1.57) ^b^	35.0	53.4	1.52 (1.44–1.61) ^b^	8.1	14.0	1.73 (1.55–1.94) ^b^	33.1	53.5	1.62 (1.53–1.71) ^b^
30–34	115.2	172.6	1.50 (1.45–1.55) ^b^	30.5	45.4	1.49 (1.40–1.58) ^b^	11.0	17.1	1.55 (1.40–1.72) ^b^	39.8	61.0	1.53 (1.45–1.62) ^b^
35–39	124.0	162.5	1.31 (1.27–1.35) ^b^	31.3	39.6	1.27 (1.19–1.35) ^b^	12.3	17.2	1.39 (1.26–1.54) ^b^	41.4	64.7	1.56 (1.48–1.65) ^b^
40–44	114.7	141.9	1.24 (1.20–1.28) ^b^	27.7	33.3	1.20 (1.12–1.29) ^b^	11.7	13.8	1.18 (1.07–1.31) ^b^	44.2	61.3	1.39 (1.32–1.46) ^b^
45–49	106.3	128.3	1.21 (1.17–1.25) ^b^	26.4	33.3	1.26 (1.18–1.35) ^b^	10.5	12.1	1.15 (1.03–1.29) ^b^	38.2	46.1	1.21 (1.14–1.28) ^b^
50–54	101.1	128.5	1.27 (1.23–1.32) ^b^	26.3	31.9	1.21 (1.13–1.30) ^b^	10.5	12.1	1.15 (1.03–1.29) ^b^	34.5	41.6	1.21 (1.14–1.29) ^b^
55–59	102.0	126.1	1.24 (1.19–1.28) ^b^	22.5	23.7	1.05 (.97–1.14)	9.9	12.5	1.26 (1.12–1.42) ^b^	34.8	43.3	1.24 (1.17–1.33) ^b^
60–64	97.2	123.3	1.27 (1.22–1.32) ^b^	15.9	17.1	1.07 (.97–1.19)	10.4	15.0	1.45 (1.28–1.64) ^b^	32.9	43.9	1.33 (1.24–1.43) ^b^
65–69	100.3	117.0	1.17 (1.12–1.22) ^b^	10.8	10.5	.97 (.84–1.12)	12.5	16.3	1.31 (1.16–1.48) ^b^	35.3	43.7	1.24 (1.15–1.33) ^b^
70–74	118.1	120.3	1.02 (.97–1.07)	9.3	9.8	1.05 (.88–1.25)	17.8	17.7	.99 (.88–1.13)	37.2	41.5	1.11 (1.02–1.21) ^b^
75–79	154.8	146.0	.94 (.90–.99) ^a^	10.0	9.2	.92 (.75–1.12)	25.3	25.2	1.00 (.88–1.13)	44.5	43.7	.98 (.90–1.08)
80–84	190.3	167.3	.88 (.83–.93) ^a^	8.7	7.3	.85 (.66–1.09)	34.2	30.9	.90 (.80–1.02)	45.4	45.0	.99 (.89–1.10)
85+	259.2	232.3	.90 (.85–.94) ^a^	7.4	4.8	.65 (.48–.89) ^a^	50.5	53.9	1.07 (.96–1.19)	61.3	53.5	.87 (.79–.96) ^a^

Source: NNDSSI, extracted on 10th October 2016

^a^ indicates significant male preponderance

^b^ indicates significant female preponderance

Note, numbers are slightly lower than total notifications due to missing data on age and sex

## Discussion

Using this large dataset (*n* = 334,560 of laboratory-confirmed influenza infections collected over the past seven years in Australia, we noted significant variations in influenza notification rates by age, sex and subtype of the virus. However, there was an overall pattern of a female predominance of influenza notifications in adult age groups, with males predominating earlier and later in life. The trends are relatively consistent across the major reporting states in Australia. The notification rate of A(H1N1)pdm09 declined with age which is consistent with the hypothesis that A(H1N1)pdm09 confers a longer lasting immunity i.e. older individuals who have been exposed to the virus would have acquired immunity [7, 9, 23, 24]. Of note, this declining notification rate was similar in both sexes. Younger individuals appear to be more susceptible to A(H1N1)pdm09 and young age is a principal mortality risk factor [8]. Health seeking behaviours may also contribute to the observed age patterns. If an older individual has acquired immunity to A(H1N1)pdm09, the individual would have less severe illness [23] and may be less likely to seek medical care. In contrast, younger individuals with more severe symptoms of influenza-like illness (ILI) caused by the virus are arguably more likely to seek medical care or be brought to medical attention. This potentially would lead doctors to investigate the cause of the illness resulting in higher laboratory-confirmed notification rates.

We found higher notification rates in females between the ages 20 and 54 years, regardless of the type or subtype of influenza, which may suggest that females in these age groups are more susceptible to influenza infection. While this observation may be confounded by the greater healthcare utilisation in women compared to men observed in Australia [25] and by targeted vaccination campaigns, the sex disparity could also be due to pregnancy-related immune-suppression. Immune responses can be suppressed during the menstrual cycle and pregnancy [10, 12–14, 19]. Inclusion of pregnancy and menstrual status in notification databases would allow for further investigation of the influence of these factors. While we found the female preponderance for A(H1N1)pdm09 was mainly during ages 15–54 years, the higher female-to-male notification rates for A (all notifications) and A(H3N2) extended to 69 years and for Influenza B to 74 years. Higher female notifications extending beyond menopause may indicate sexual dimorphisms that pertain to the virus subtype, immune response genes, sex chromosome and hormonal differences [26]. Health-seeking behaviours and vaccination campaigns are independent of influenza virus type and subtype, so cannot account for the differences in notification rates observed across virus subtypes in peri- and post-menopausal women.

Regardless of virus subtype, male notification rates were always higher than female rates for those aged 0 to 4 years. Muenchhoff and colleagues [27] reported that pre-pubertal boys are more susceptible to viral infections possibly due to their shorter upper airways [28] which could increase their susceptibility to respiratory tract infections. Muenchhoff and colleagues also acknowledge that the effect of different sick-role behaviours between boys and girls at different ages requires further research [27]. Male dominance in notifications was also observed among males aged 85 years and above who had Influenza A, A(H1N1)pdm09 and Influenza B.

Vaccination status affects influenza infection and female-to-male notification rates. While Klein and colleagues reported that females were less likely to accept vaccines [13], data from Australia indicate greater national coverage in females (76.5%) than males (72.2%) aged 65 years and older in 2009 [29]. Data also indicate increased coverage by age, with overall coverage rates in 2014 ranging from 24% among 18 to 24 years old up to 73% in those aged 65 years and older [30]. However, as individual vaccination status is not included in the Australian national surveillance database, we cannot determine the impact of vaccination on notification rates in this study.

There are several other social and lifestyle factors, beyond sickness behaviours and vaccinations status, that may influence virus exposure and thus notification rates. For example, female preponderance might be explained by increased contact with young children. There is some recent evidence that obesity, with its consequent immune dysfunction, may impact not only on the severity of influenza illness, but also on transmission, with increased viral shedding duration for Influenza A virus [31]. Similarly smoking has been associated with more frequent and severe influenza infection, and to reduce vaccine efficacy in the elderly [32]. Social determinants of health such as education and socioeconomic status can also impact on rates of infection [33]. In turn, each of these factors may differ between females and males and account for the observed gender differences in relative rate ratios.

We note that the NNDSSI Influenza Public Dataset does not include data from all regions of Australia (the Australian Capital Territory is not included) and 78.1% of the data were without identification of Indigenous status, thus rendering the variable unavailable for analysis. Furthermore, the denominators (estimated resident populations) used in all analyses were obtained from a secondary source, specifically the Australian Bureau of Statistics. While unreliability of these population data could contribute to errors in the notification rates and hence the relative rate ratios, we postulate that the errors (should they exist) would be random and not influence the observations of gender disparity by types and subtypes of the influenza viruses. Another possible study limitation results from the predominance of the A(H1N1)pdm09 sub-type during the 2009 pandemic, which focussed laboratory testing on this subtype and consequently notification rates may be inflated for 2009 and the following few years. However, we note that the A(H1N1)pdm09 notification rates and female: male ratios were similar for 2009 and 2010–2015.

In addition, notifications data under-represent cases [34]. The NNDSSI also note that changes in disease prevalence or incidence may reflect changes in testing policies and methods, targeted screening programs and influenza awareness campaigns. However, our observations remain robust since we would not expect to observe variation by virus type and subtype in the cases under-represented in notifications or by the impact of changes in policy.

## Conclusions

There are significant age and sex disparities in Influenza notifications among various types of influenza in Australia. The A(H1N1)pdm09 notification rate declined with age in both sexes, consistent with the hypothesis that the longer lasting immunity protects older populations who have had prior exposure to the virus. The differences observed in peri- and post-menopausal women across virus subtypes are unlikely to be affected by different health-seeking behaviours or targeted vaccination campaigns. Higher female notifications extending beyond menopause suggests that there is an interaction between age and sex on susceptibility to influenza infection which varies by the subtype of the virus. Public health authorities would need to establish systems to include recording of pregnancy, menopausal, and vaccination status of individuals in order to allow analysis of these factors with potential to affect an individual’s susceptibility to influenza infection. Collaborative international efforts in these regards may lead to future studies on the relative merits of age and sex specific influenza vaccines.
